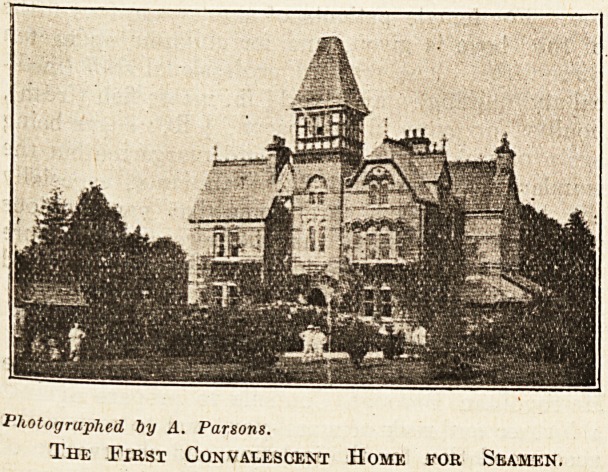# Hospital and Institutional News

**Published:** 1918-11-09

**Authors:** 


### HOSPITAL AND INSTITUTIONAL NEWS.

### THE "FIRST CONVALESCENT HOME FOR SEAMEN."

MRS. ANGAS, of Dirry Mor, Stoke Poges, has given to the Seamen's Hospital, Greenwich, in recognition of the country's indebtedness to the sailor, a convalescent home on the North Kentish Downs. The generous gift is in response to Sir Rosslyn Wemyss' advocacy of the claims of seamen. This is the first time that the seaman, to whom England owes its freedom from invasion, has been provided with a Home where he can rest awhile when recovering from wounds or accident on the high seas, men who have been torpedoed (many over and over again), convalescents from tropical diseases, and the shipwrecked man exhausted by exposure and exhaustion. All these will be able

*Photographed by A. Parsons.*

THE FIRST CONVALESCENT HOME FOR SEAMEN.

to recuperate, after treatment in the "Dreadnought," before returning to life afloat. The Home is freehold, and is close to the small village of Cudham, 600 feet above sea-level, and within six miles of Sevenoaks. It is surrounded by 18 acres of meadow-land and gardens, and will accommodate thirty sailors. It will cost about £2,500 per annum to maintain. The sailor, always out of sight and often forgotten, is at last remembered, and towards the responsibility of maintenance valuable assistance has been given, as will be seen, by Major R. M. Everett (£2,500); and Mrs. Langford has endowed a bed by a gift of £1,000. An anonymous friend of the sailor has promised £250 on condition that nine other donations of a like amount are forthcoming. Mrs. E. Spencer Stidolph, of Langdale House, Greenwich, has given one of the needed sums.

### LADY MARKHAM'S EXAMPLE.

THERE is a debt of £5,000 on the Mansfield and District Hospital, and the Board of Management, with the aid of the local press, is trying to raise forty thousand half-crowns before the beginning of next year. Lady Markham, of Newstead Abbey, hoping that people may be persuaded to endow beds, has handed to the hospital a cheque for £1,000. This will endow the first bed, in the name of her late husband, Sir Arthur Markham, Bart, M.P.

### FOOD INSPECTION AND STANDARD SHIPS.

DR. W. M. WILLOUGHBY, medical officer for the Port of London, provides in his annual report some statistics regarding the inspection of food into London which are extremely interesting considering the difficulties of transport at the present time. Last year the seizures amounted to 4,847 tons, as against 5,664 tons in 1916, 3,118 in 1915, and 1,185 in 1914. More than half of the total quantity represents grain. In 1916 the seizures of tea and coffee respectively were 400 tons and 2[illegible]4 tons, but last year not an ounce of tea failed to pass the test, and only one ton and a quarter of coffee had to be condemned These figures are remarkable, in view of the present transport difficulties, and may be accounted for either by the adoption of a lower standard or by more careful package for transport. Ten years ago the cost of administration of this department of the City's activities was only between £8,000 and £10,000, but last year the figures went up to £15,454. Dr. H. Williams, the late medical officer, pioneered sanitary reform on board ship, but unfortunately did not live to see the adoption of his improvements. The building of the so-called standard ship has afforded the opportunity desired. In the latest report of the medical officer it is shown that these improvements have been partly carried out by the berthing of the crew in the poop instead of the forecastle, the provision of two separate cubicles, the allotment of separate messing, and the increase of the cubic space per man. The effect can only be to raise the standard of health on board ship.

### A GOOD WEEK FOR LEGACIES.

THE estate of Mrs. Jessie Smith, of Glasgow, the widow of Mr. Finlay Smith, a tobacco manufacturer, amounts to £233,726, and handsome provision has been made for Scottish hospitals and other charities after the expiry of a life rent. £10,000 will go to Glasgow Royal Infirmary to endow a ward in memory of the late husband of the testatrix, and £5,000 each to the Western and Victoria Infirmaries. There are many other deferred legacies, amongst which one of £2,000 to the Royal Samaritan Hospital for Women and another of equal amount to Ayr County Hospital. The Medical School of Guy's Hospital will benefit largely under the will of the late Dr. Louis A. Dunn, F.R.C.S., of Gerrard's Cross, after the death of his two brothers. The gross value of the estate is £68,335, and apparently the whole of this important sum will eventually be devoted to medical education. Dr. Dunn, who also leaves to Guy's the gold medal presented to him by London University when he took his degree as Master in Surgery, was consulting surgeon to a number of institutions, including St. Mary's Hospital, Plaistow.

### A LIGHTERMAN'S THANK-OFFERING.

KING'S COLLEGE HOSPITAL has received a munificent gift of £10,000 to endow a ward for working-men, to be named "The George Trundle Ward." The donor, Mr. George Trundle, who has handed over to the hospital authorities securities to the value of £10,000 for that purpose, was for many years occupied as a lighterman at Bankside, and he is making this gift as a thank-offering for his successful business career. Its form is one which he feels calculated to benefit his fellow-men in a greater degree than any other, and the hospital committee cannot fail to be encouraged by Mr. Trundle's generosity in the effort they are making to raise £100,000 to carry on their work.

### AN APPEAL TO THE COCK AND HEN CLUB.

THE deadlock over the laundry between the Board of Control and the Joint Counties' Mental Hospital at Carmarthen still continues. A new laundry was built more than a year ago, but since the Board of Control withheld permission for the purchase of the necessary machinery, no use has been made of it. As long ago as October 29, 1917, the Visiting Commissioner of the Board of Control, in reporting on the laundry, said that "the continuance of the present state of things can only be justified by the abnormal conditions produced by the war, and it would be of great benefit to the asylum if public requirements of more vital importance do not preclude the immediate carrying out of the improvement" That was twelve months ago, and still the laundry is minus the necessary machinery. Strong protests against the continuance of the Board of Control's refusal were made at a recent meeting of the Asylum Visitors, when it was decided to appeal to the six M.P.s representing the constituent counties—Carmarthen, Cardigan, and Pembroke—to raise the matter in the latest "cock and hen" club, the House of Commons.

### ANTI-TUBERCULOSIS PROPAGANDA IN FRANCE.

WHETHER the present troubled state of France makes it a very good time for public health propaganda there to be undertaken by a travelling commission of foreigners may perhaps be doubted. However, with the principles laid down at the recent annual meeting of the National Association for the prevention of consumption by an American, Dr. Gunn, there can be no quarrel. They are the well-worn arguments now familiar and accepted. The methods of inculcating them upon the lay public have also been standardised—the magic-lantern slides, serious and occasionally comic, the film, the health chat column in the newspaper, the lecture, the travelling exhibition, the poster, the pamphlet, and so forth. This sort of work seems to have been done in some nine departments of the country of Laënnec, and as the French profession has probably got its hands full with other work, they may have been glad of the assistance of their American *confrères*, who came under the auspices of the Rockefeller Foundation. It is often forgotten that France is credited on the Continent with being the home of the dispensary movement, but one can never have too much of a good thing, and to hear that others also believe in sanitation will have awakened the always polite attention of the French. It must be eight or nine years ago that we reviewed in THE HOSPITAL one of the completest schemes for a colony ("Le Sanatorium Village") for the tuberculous that has ever been drawn up. After the war, French doctors should return the call, and preach the advantages of National Insurance and Workmen's Compensation Acts.

### FOOD RATIONS IN SANATORIA.

THE Food Controller and the President of the Local Government Board have revised the dietary scale for tuberculosis residential institutions which was set up in April. For male patients over ten years the amounts are very satisfactory—4 lb. meat, 1 lb. fish and poultry, ½ lb. bacon weekly, although sugar is still rather short, being only ½ lb. Butter and margarine are only twice the usual allowance too. For female patients of similar age four-fifths of the above is given, and for children under ten three-fifths. The resident professional staff (medical and nursing) may have 1¼ lb. meat; fish (fresh), poultry, or game, 2 lb.; bacon, ½ lb.; sugar being again only ½ lb., and butter and margarine but the standard five ounces. These amounts, especially the last two items, are somewhat parsimonious when it is remembered that the necessary exposure to cold of sanatorium residents causes them to need plenty of heat-forming food.

### THE TREATMENT OF SMALL-POX CONTACTS.

THE London County Council has warned the Metropolitan Borough Councils to prepare against a further outbreak of small-pox, and to review the accommodation for the isolation and observation of contacts. In Camberwell, Dr. Francis Stevens, the medical officer of health, reported that all contacts were visited daily for fourteen days after possible exposure to infection, and in view of this fact the Council is not in favour of providing shelters, which are found costly and unnecessary. The London County Council, however, were not convinced, and in a further report Dr. Stevens states that everyone would agree to keep contact cases under observation, but he denied the necessity for isolating them. So far as he was aware, there was no evidence that the disease was infectious during incubation, and by the inspector's daily visits to houses where cases had occurred, suspicious symptoms could be notified. This plan was more practical than the shelter, which lost even theoretical value without a resident medical man.

### SOUTHWARK TRIBUNAL AND THE STAFF AT GUY'S HOSPITAL.

DR. M. S. PEMBREY again had to appear before the Southwark Tribunal to appeal on behalf of the governors of Guy's Hospital for the further exemption from military service of the chief assistant in the laboratory and operating theatre. He was thirty-two years of age, and was in Grade 1, and unfortunately his age precluded him from coming within the scope of the new list of certified occupations. Dr. Pembrey pointed out that the applicant's long experience at the hospital, lasting over eighteen years, was invaluable, and that, should he have to go, his duties in preparing for the lectures would have to be performed by one of the greatly reduced medical staff. Since the outbreak of war, he added, over ninety men from Guy's had given their lives. To cope with the shortage in the medical profession they had been compelled to open their medical school a month earlier than usual, and students were now allowed to begin their studies at sixteen years of age. This concession was due to the action taken by the Ministry of National Service. The Tribunal granted a further four months' exemption. It is to be regretted that the chief assistant did not come within the limits of the new certified occupation list, so that Guy's Hospital might have avoided further appeals for his retention.

### THE HOSPITAL POSTER AT BOURNEMOUTH.

At last the hospital poster, for which we have argued often, is coming into its own. The excellent financial result of the recent Alexandra Rose Day held in Bournemouth, largely due to this, was announced in the Council Chamber, when, on behalf of the Bournemouth Rose Day Committee, Councillor Fox, its chairman, formally presented the Mayor, as president of the committee, with a cheque for £1,000 for the Royal Victoria and West Hants Hospital. The committee for some years has fixed its goal at £1,000, but 1918 is the first year in which this total has been reached. The Mayor congratulated Councillor Fox and his committee upon having achieved their aim by raising over £1,000, and said that it was gratifying to know that the money was going to the Royal Victoria and West Hants Hospital. Mr. Godwin Pratt, the honorary treasurer of the hospital, in accepting the cheque, also congratulated the committee as only a treasurer can. The success of the hospital, he said, depended upon the appreciation of the institution by the public which supported it. The interesting fact was mentioned during the afternoon that the posters designed by the Bournemouth Municipal School of Art for the committee were so successful that they had been adopted by the London Central Committee for general use throughout the country.

### "NO FIRES IN THE BEDROOMS": AN ALTERNATIVE PROPOSAL.

The governing bodies of hospitals and other institutions are energetically taking in hand saving in coal and light consumption. After some consideration, the Edmonton Board of Guardians has passed a resolution to the effect that "no fires shall be lit in the bedrooms of any of the staff at the hospital, infirmary, and other institutions of the board except by the direction of the medical officer." Any fires so used must be the subject of a report to the next meeting of the Edmonton Guardians. Whilst saving in fuel consumption is very necessary this winter, it is to be hoped that this rule will not be administered too rigidly, so as to occasion hardship to a body of hard-working women. Although we are all becoming used to being "rationed," the alternative might be considered of making the staff a "bedroom allowance," which would be appreciated by the nurse who, though not ill, desires the restful privacy of her own room occasionally.

### THE AMERICAN HOSPITAL IN WALES.

Through the generous action of Major J. W. Beynon, J.P., who has handed over that big house, The Coldra, at Christchurch, near Newport (Mon.), and its grounds of 120 acres, to the American Red Cross for the duration of the war and a period afterwards, workmen are now engaged in preparing there one of the largest American hospitals in Great Britain. The house itself is being transformed for the reception of about 100 beds, with accommodation for the staff, and huts are to be erected in the surrounding grounds for several hundred additional cases.

### STATE PRISONERS IN HOLLAND.

A Dutch correspondent writes: At Woensel, in the immediate neighbourhood of Einthoven, in Holland, has been opened lately a new State asylum for the care of lunatic State prisoners. It includes eight pavilions (five for men and three for women) for 100 patients each; a very modern-built "fortified department" for the very turbulent and dangerous patients; a great administrative building, with laboratories, *x*-ray department, medical, surgical, and physical equipment, etc. This asylum has its own installation for electric lighting, heating with hot-water pipes, central kitchen, laundry, and also a church. Worthy of mention are the carpenters', painters', shoemakers', tailors', and matting-makers' workshops as establishments for procuring employment for the patients. For that purpose serves also the farmwork on the grounds of the asylum, rather more than 325 acres in size. The budget for the building was two years ago near £160,000, but in the event the asylum has actually cost upwards of £300,000. Dr. Reitsma has been appointed as physician superintendent.

### THE PRIVATE NURSING OF LUNATICS IN HOLLAND.

In accordance with the experience of the Gheel colony in Belgium and in Veldwijk, near Ermeloo, there is in Holland an increasing belief in the nursing of lunatics in families, as opposed to the institutional care of the insane. To encourage this attempt the late Royal Medical Inspector of the Dutch asylums, Dr. S. van Deventer, has left a legacy to establish a special fund for this purpose. This fund is named the "Antonia-Wilhelmina Fund," after the late wife of Dr. van Deventer. Under the old Dutch Lunacy Act of 1884 there was only one type of asylum, the "closed" one. For the confinement of a patient in such an asylum one needed always not only a medical certificate but also a judicial mandate. All this was to protect sane patients from the improper intentions of their families. With the same Act the Royal medical inspection of asylums was instituted, but the aftercare of the insane was greatly hampered through the necessity of certification. The present Royal Medical Inspector of Asylums, Dr. J. H. Schuurmans Stekhoven, sought an opportunity to improve the treatment of the patients. He found it in the establishment of "open" asylums. The Dutch Government has accepted the advantages of this new system and not hesitated to encourage the establishment of open wards and open departments in the old and the recently built asylums, where voluntary boarders are now accepted.

### NATIONAL ASSOCIATION FOR PREVENTION OF CONSUMPTION.

The report submitted to the nineteenth annual meeting of the above body recorded encouraging results of the appeal made last year for £50,000 to start a farm colony for discharged tuberculous sailors and soldiers. Of this large sum £24,000 had been collected, including donations from three members of the Royal Family. A site for the colony has been chosen at Frimley, close to the well-known sanatorium of Brompton Hospital. On 118 acres of land there temporary buildings have been erected, and the ground begun to be cleared. It is hoped to receive the first colonists in the spring. These men will have already had a course of sanatorium treatment, and will be in a condition to do a full day's work, which may include nearly every branch of agriculture. It is hoped also to give support to the Farm Colony at Polton, near Edinburgh. Action is contemplated in setting up small local hospitals for advanced cases of consumption in different places, the idea being that patients in such a state will not go far from home, and dislike big institutions. This is undoubtedly the case. During the year the Council of the Association sustained a great loss through the death of Mr. Alfred de Rothschild, one of its treasurers.

### THE "WAR BEETLE" BED AT BELFAST.

The men employed at Messrs. Workman, Clark and Co.'s shipyard at Belfast recently completed the *War Beetle*, a standard ship, in 3¾ days from the time of its launch. The firm was anxious that this smart piece of work should be fittingly acknowledged and asked the men what form it should take. Their appreciation of the Royal Victoria Hospital decided the men to refuse personal gifts and to suggest the endowment of a bed at that institution. The firm have therefore sent a cheque for £250 to endow a bed at the hospital for five years in the name of the foremen, leading hands, and workmen engaged on the *War Beetle*.

### THE CHEERFUL COLLIE.

Pound Day at the Anti-Vivisection Hospital, Battersea Park, was a success, for altogether 400 pounds of goods were received. Some must have entailed real sacrifice, for they included pounds of rationed articles difficult to obtain, which are essential in the home, especially where there are young children. The cash subscribed amounted to about £80, which compares favourably with former totals. A cheerful collie with a collecting-box obtained £33. All contributors of one pound in cash or goods were allowed freely to inspect the hospital, where they were met by Lord Tenterden and the Mayor and Mayoress of Battersea.

### NEW PHARMACIST FOR THE BROMPTON HOSPITAL.

The vacancy caused by the death of Mr. John Hammond Crane, who was for many years chief pharmacist at the Brompton Hospital for Diseases of the Chest, has now been filled. The successful candidate is Mr. Francis Henry Gillett, who has been on the dispensary staff at the London Hospital under Mr. F. Almond Hocking, B.Sc. (Lond.). Mr. Gillett is a member of the Pharmaceutical Society, and holds the "Major" diploma in pharmacy granted by that body. We understand that several ladies were candidates for the post; but evidently the unique experience gained by several years' work at the "London" dispensary carried weight with the selection committee of the Brompton Hospital.

### WHAT IS AN "APPLIANCE"?

An interesting question is raised as to the meaning of an "appliance" by the following incident which has recently occurred:—A clergyman of a contributing parish applied to the Hospital Sunday Fund for a grant of dressings—absorbent wool and gauze—for a woman who had recently undergone a severe operation. The Hospital Sunday Fund replied that to its great regret it was unable to help the case as its funds were available only in this direction for "surgical appliances." The dictionary defines "apply" as "to lay on," or "put on." Are dressings, then, applied internally?

### HOW TO DO IT.

Congratulations are due to the Newcastle Infirmary authorities, who, by their energetic efforts, had succeeded in raising by the end of last month £45,000 towards the establishment of an orthopædic centre at Newcastle. They thus become entitled to a Government grant of £10,000, which was promised upon the condition that the sum now realised should be raised locally.

### THIS WEEK'S DRUG MARKET.

The character of the market is much the same as last week; that is to say, there is very little purchasing of drugs that are not immediately required, but considerable buying of drugs in vogue for the treatment of influenza. English refined camphor is dearer. Eucalyptus oil continues to be in good demand, and prices are firmly maintained. Aspirin is selling well and the price still tends upwards. At the time of writing the fixed prices for quinine had not been announced, and in the meantime the demand is good and values are maintained. Atropine sulphate is offered at lower prices. Opium tends upwards in value. Boric acid is dearer. There is a strong demand for cinnamon oil, and very high prices are asked.

## Figures and Tables

**Figure f1:**